# Associations of cognitive performance with cardiovascular magnetic resonance phenotypes in the UK Biobank

**DOI:** 10.1093/ehjci/jeab075

**Published:** 2021-05-14

**Authors:** Zahra Raisi-Estabragh, Amine M'Charrak, Celeste McCracken, Luca Biasiolli, Maddalena Ardissino, Elizabeth M Curtis, Nay Aung, Claudia K Suemoto, Clare Mackay, Sana Suri, Thomas E Nichols, Nicholas C Harvey, Steffen E Petersen, Stefan Neubauer

**Affiliations:** Centre for Advanced Cardiovascular Imaging, William Harvey Research Institute, NIHR Barts Biomedical Research Centre, Queen Mary University of London, Charterhouse Square, London EC1M 6BQ, UK; Barts Heart Centre, St Bartholomew’s Hospital, Barts Health NHS Trust, London EC1A 7BE, UK; Division of Cardiovascular Medicine, Radcliffe Department of Medicine, University of Oxford, National Institute for Health Research Oxford Biomedical Research Centre, Oxford University Hospitals NHS Foundation Trust, Oxford, OX3 9DU, UK; Centre for Advanced Cardiovascular Imaging, William Harvey Research Institute, NIHR Barts Biomedical Research Centre, Queen Mary University of London, Charterhouse Square, London EC1M 6BQ, UK; Division of Cardiovascular Medicine, Radcliffe Department of Medicine, University of Oxford, National Institute for Health Research Oxford Biomedical Research Centre, Oxford University Hospitals NHS Foundation Trust, Oxford, OX3 9DU, UK; Division of Cardiovascular Medicine, Radcliffe Department of Medicine, University of Oxford, National Institute for Health Research Oxford Biomedical Research Centre, Oxford University Hospitals NHS Foundation Trust, Oxford, OX3 9DU, UK; Faculty of Medicine, Imperial College London, London SW7 2AZ, UK; MRC Lifecourse Epidemiology Unit, University of Southampton, Southampton, UK; Centre for Advanced Cardiovascular Imaging, William Harvey Research Institute, NIHR Barts Biomedical Research Centre, Queen Mary University of London, Charterhouse Square, London EC1M 6BQ, UK; Barts Heart Centre, St Bartholomew’s Hospital, Barts Health NHS Trust, London EC1A 7BE, UK; Division of Geriatrics, Department of Internal Medicine, University of Sao Paulo Medical School, Sao Paulo, Brazil; Department of Psychiatry, University of Oxford, Oxford, UK; Department of Psychiatry, University of Oxford, Oxford, UK; Nuffield Department of Population Health, Oxford Big Data Institute, Li Ka Shing Centre for Health Information and Discovery, University of Oxford, Oxford OX3 7LF, UK; Nuffield Department of Clinical Neurosciences, Wellcome Centre for Integrative Neuroimaging, FMRIB, University of Oxford, Oxford OX3 9DU, UK; MRC Lifecourse Epidemiology Unit, University of Southampton, Southampton, UK; NIHR Southampton Biomedical Research Centre, University of Southampton and University Hospital Southampton NHS Foundation Trust, Southampton, UK; Centre for Advanced Cardiovascular Imaging, William Harvey Research Institute, NIHR Barts Biomedical Research Centre, Queen Mary University of London, Charterhouse Square, London EC1M 6BQ, UK; Barts Heart Centre, St Bartholomew’s Hospital, Barts Health NHS Trust, London EC1A 7BE, UK; Division of Cardiovascular Medicine, Radcliffe Department of Medicine, University of Oxford, National Institute for Health Research Oxford Biomedical Research Centre, Oxford University Hospitals NHS Foundation Trust, Oxford, OX3 9DU, UK

**Keywords:** cardiovascular magnetic resonance, cardiovascular disease, brain, cognition, dementia, heart–brain axis, vascular risk factors

## Abstract

**Aims:**

Existing evidence suggests links between brain and cardiovascular health. We investigated associations between cognitive performance and cardiovascular magnetic resonance (CMR) phenotypes in the UK Biobank, considering a range of potential confounders.

**Methods and results:**

We studied 29 763 participants with CMR and cognitive testing, specifically, fluid intelligence (FI, 13 verbal-numeric reasoning questions), and reaction time (RT, a timed pairs matching exercise); both were considered continuous variables for modelling. We included the following CMR metrics: left and right ventricular (LV and RV) volumes in end-diastole and end-systole, LV/RV ejection fractions, LV/RV stroke volumes, LV mass, and aortic distensibility. Multivariable linear regression models were used to estimate the association of each CMR measure with FI and RT, adjusting for age, sex, smoking, education, deprivation, diabetes, hypertension, high cholesterol, prior myocardial infarction, alcohol intake, and exercise level. We report standardized beta-coefficients, 95% confidence intervals, and *P*-values adjusted for multiple testing. In this predominantly healthy cohort (average age 63.0 ± 7.5 years), better cognitive performance (higher FI, lower RT) was associated with larger LV/RV volumes, higher LV/RV stroke volumes, greater LV mass, and greater aortic distensibility in fully adjusted models. There was some evidence of non-linearity in the relationship between FI and LV end-systolic volume, with reversal of the direction of association at very high volumes. Associations were consistent for men and women and in different ages.

**Conclusion:**

Better cognitive performance is associated with CMR measures likely representing a healthier cardiovascular phenotype. These relationships remained significant after adjustment for a range of cardiometabolic, lifestyle, and demographic factors, suggesting possible involvement of alternative disease mechanisms.

## Introduction

Cardiovascular disease and cognitive impairment are growing public health problems, particularly in ageing global populations.^1^^,^^2^ Existing work suggests interactions across heart–brain organ systems. The brain has been proposed as a target for end-organ damage from cardiovascular disease and risk factors.^3^ Indeed, cardiometabolic morbidities have been linked to accelerated cognitive decline^4^^,^^5^ and their treatment with slowed progression of dementia.^6^ Cardiovascular risk factors have been associated with both vascular^7^ and Alzheimer’s dementia.^5^ In individuals without dementia, vascular risk factors correlate with worse cognitive performance, with an additive effect from increasing number of risk factors.^8^ Furthermore, cardiovascular risk factors are associated with poorer brain health across grey and white matter macrostructure and microstructure assessed on brain magnetic resonance imaging.^9^

There is support for common heart–brain disease pathways mediated by atherosclerosis and arteriosclerosis.^3^ However, the precise mechanisms by which cardiovascular diseases and risk factors may cause cognitive impairment are incompletely understood, and it is not known if alternative mechanisms may play a role in the observed associations. Exploring the relationship between cognitive performance and indices of cardiovascular structure and function may provide novel insights into these relationships and their underlying mechanisms; however, to date, this has not been studied in large cohorts.

We studied, in the UK Biobank, associations of cardiovascular magnetic resonance (CMR) indices of cardiovascular structure and function with cognitive performance measures. We considered potential confounding from a wide range of cardiometabolic, lifestyle, and demographic exposures.

## Methods

### Study population and setting

The UK Biobank is a large prospective cohort study incorporating data from over half a million participants from across the UK. Individuals aged 40–69 years old were identified through National Health Service (NHS) registers and recruited over a 4-year period between 2006 and 2010 from a range of urban and rural settings.^10^ The protocol is publicly available.^11^ Baseline assessment comprised detailed characterization of sociodemographics, lifestyle, environmental factors, medical history, tests of cognitive function, and a series of physical measures. Individuals who were unable to consent or complete baseline assessment due to illness or discomfort were not recruited. The UK Biobank Imaging Study, which aims to scan 100 000 of the original participants (48 000 completed, February 2021),^12^ includes, among a wide range of other assessments, detailed CMR imaging.^13^

### Ethics

This study was covered by the ethical approval for UK Biobank studies from the NHS National Research Ethics Service on 17 June 2011 (Ref 11/NW/0382) and extended on 10 May 2016 (Ref 16/NW/0274). All participants provided written informed consent.

### Measures of cognitive function

We assessed cognitive measures available in terms of biological relevance and repeatability. We selected two components from the UK Biobank cognitive function assessment for inclusion in our analysis: fluid intelligence (FI) and reaction time (RT). The FI test is intended to measure the capacity to solve problems that require logic and reasoning independent of acquired knowledge. The RT exercise is designed to provide a crude measure of raw processing speed, reaction speed, and attention. Overall, these two tests provide broad assessment of several different aspects of cognitive performance. Additionally, these are robust measures, with demonstrated reliability (internal consistency) and longitudinal stability in previous work.^14^^,^^15^ Furthermore, their availability for a large subset of the UK Biobank imaging cohort permits adequately powered analyses of associations with CMR imaging phenotypes.

### Fluid intelligence

Assessment of FI consisted of a series of 13 verbal-numeric reasoning questions completed within 2 minutes. A point was awarded for each correct answer; incorrect, or unanswered questions received a score of zero. The final score was the sum of correct answers with a maximum score of 13. Thus, higher FI scores correspond to higher cognitive performance. The Cronbach alpha reliability for this test is 0.62.^14^ The full protocol for FI assessment in UK Biobank is published elsewhere^16^; we provide a summary of the questions in Supplementary data online, *Table S1*. As the FI variable in our sample was normally distributed, it was treated as a continuous numerical variable for purposes of modelling, as per established methods.^17^

### Reaction time

The RT test consisted of four rounds of a pairs matching exercise. In each round, participants were shown 12 pairs of cards on a screen and asked to press a button as soon as a matched pair of cards appeared. The final RT score is calculated as the mean time in milliseconds to correctly identify matches over four rounds. Hence lower RT scores represent faster processing speed and better cognition. The Cronbach alpha reliability for this test is 0.85.^11^ The full protocol for the RT test is available in a dedicated document.^18^

### CMR image acquisition and analysis

UK Biobank CMR scans are performed using 1.5 T scanners (MAGNETOM Aera, Syngo Platform VD13A, Siemens Healthcare, Erlangen, Germany) according to a standardized protocol.^19^ Assessment of the left and right ventricles (LV and RV) includes a complete short-axis stack acquired using balanced steady-state free precession sequences. Conventional LV and RV volumetric measures were extracted using a fully automated quality controlled pipeline previously developed and validated in a large subset of the UK Biobank, as detailed elsewhere.^20^ Aortic distensibility represents the relative change in area of the aorta (aortic strain) per unit pressure. Aortic strain was measured using transverse cine images of the aorta and divided by central pulse pressure from Vicorder^®^ readings at the time of imaging. Aortic distensibility results were obtained from a previous analysis of UK Biobank scans using a purpose-designed automated quality controlled tool.^21^ Thus, we considered the following CMR measures: LV/RV volumes in end-diastole and end-systole, LV/RV ejection fraction, LV/RV stroke volume, LV mass, and aortic distensibility at the proximal descending aorta.

### Statistical analysis

Statistical analysis was performed using R version 3.6.2^22^ and RStudio Version 1.3.1093.^23^ We included all participants with CMR and at least one of FI or RT. Participants with dementia, ascertained from UK Biobank algorithmically defined health outcomes, were excluded (*n* = 13). We tested, in individual multivariable linear regression models, the association of CMR metrics with measures of cognitive performance (FI and RT). Based on existing literature and biological plausibility, we considered the following covariates, determined a priori: age, sex, smoking, alcohol intake, exercise level, education, deprivation, diabetes, hypertension, high cholesterol, and prior myocardial infarction. There was no evidence of multicollinearity based on a conservative variance inflation factor threshold of <2. For ease of interpretation and to allow comparison of magnitude of effects across CMR measures, we report standardized beta-coefficients with corresponding 95% confidence intervals and *P*-values. Thus, results are standard deviation change in FI/RT per 1 SD increase in CMR measure. *P*-values are adjusted by Benjamin Hochberg method, where all CMR-related *P*-values across the set of models are adjusted together, setting a conservative false discovery rate of <5%.^24^ We performed sex-stratified analyses and tested for interaction effects by age and sex. All models were assessed for potential non-linearity using squared and cubic polynomial terms.

### Ascertainment of covariates

We used age at the imaging visit. Sex was taken as recorded at baseline. Educational level, smoking status (current vs. never/previous), and alcohol intake (intake frequency) were based on self-report. Material deprivation is reported as the Townsend index, a measure of material deprivation relative to national averages.^25^ A continuous value for the amount of physical activity measured in metabolic equivalent (MET) minutes/week was calculated by weighting different types of activity (walking, moderate, or vigorous) by its energy requirements using values derived from the International Physical Activity Questionnaire study.^26^ Diabetes was coded based on self-report of the diagnosis, self-reported use of ‘medication for diabetes’, or serum glycosylated haemoglobin >48 mmol/mol. Hypertension was coded based on self-report of the diagnosis or self-reported use of ‘medication for high blood pressure’. High cholesterol was coded based on self-report of the diagnosis, self-reported use of ‘medication for high cholesterol’, or serum total cholesterol >7 mmol/L. Prior myocardial infarction was ascertained from UK Biobank algorithmically defined outcome data.^27^

## Results

### Baseline population characteristics

**Figure 1:**
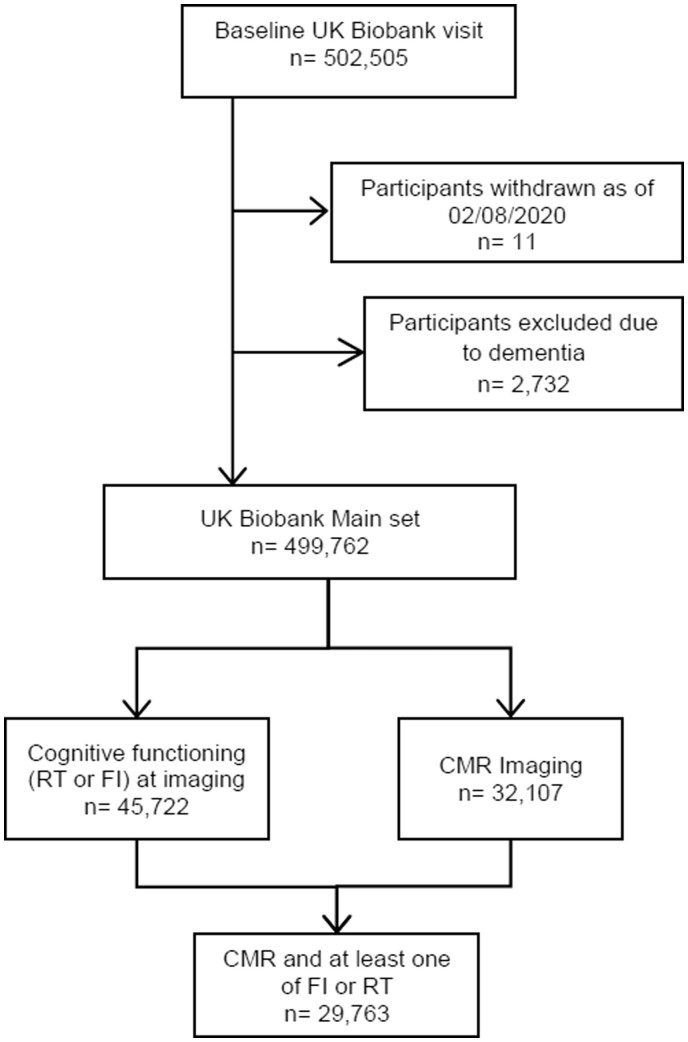
Flowchart of UK Biobank participants included in the analysis. CMR, cardiovascular magnetic resonance; FI, fluid intelligence; RT, reaction time.

There were 32 107 participants with CMR measures and without dementia, of these, FI and RT were available for 29 243 and 29 683 participants, respectively. Overall, there were 29 763 participants with CMR data and at least one cognitive function measure (*Figure 1*). The analysis sample comprised 14 379 men and 15 384 women. Mean age was 63.0 (±7.5) years. Rates of diabetes, hypertension, high cholesterol, and smoking were 3.0%, 13.5%, 22.3%, and 6.2%, respectively, with greater burden in men (*Table 1*). Overall, the analysis sample was healthier and more affluent than UK national averages. Average FI and RT were 6.7 (±2.1) items and 573 (518, 644) ms, respectively, as measured at the imaging visit.

**Table 1:** Baseline population characteristics

	**Whole cohort (*n* = 29** **763)**	**Men (*n* = 14** **379; 48.3%)**	**Women (*n* = 15** **384; 51.7%)**
Age at imaging	63.0 (±7.5)	63.7 (±7.6)	62.4 (±7.3)
Current smoker	1851 (6.2%)	1066 (7.4%)	785 (5.1%)
Education			
Left school age ≤14 years without qualifications	75 (0.3%)	42 (0.3%)	33 (0.2%)
Left school at age ≥15 without qualifications	1981 (6.7%)	954 (6.6%)	1027 (6.7%)
High school diploma or equivalent	3900 (13.1%)	1500 (10.4%)	2400 (15.6%)
Sixth form qualification or equivalent	1691 (5.7%)	751 (5.2%)	940 (6.1%)
Professional qualification (e.g. teaching, nursing)	8283 (27.8%)	4198 (29.2%)	4085 (26.6%)
Higher education university degree	13 526 (45.4%)	6782 (47.2%)	6744 (43.8%)
Townsend score	−2.7 (−3.9, −0.7)	−2.7 (−4.0, −0.7)	−2.6 (−3.9, −0.6)
IPAQ (MET minutes/week)	1530 (671, 3016)	1590 (693, 3111)	1464 (642, 2933)
Alcohol intake			
Daily or almost daily	6554 (22.0%)	3832 (26.6%)	2722 (17.7%)
Three or four times a week	8426 (28.3%)	4388 (30.5%)	4038 (26.2%)
Once or twice a week	7731 (26.0%)	3632 (25.3%)	4099 (26.6%)
One to three times a month	3223 (10.8%)	1227 (8.5%)	1996 (13.0%)
Special occasions only	2423 (8.1%)	717 (5.0%)	1706 (11.1%)
Never	1390 (4.7%)	574 (4.0%)	816 (5.3%)
Diabetes	893 (3.0%)	581 (4.0%)	312 (2.0%)
Hypertension	4016 (13.5%)	2417 (16.8%)	1599 (10.4%)
High cholesterol	6640 (22.3%)	3616 (25.1%)	3024 (19.7%)
Prior MI	590 (2.0%)	494 (3.4%)	96 (0.6%)
Fluid intelligence (items)	6.7 (±2.1)	6.8 (±2.1)	6.5 (±2.0)
Reaction time (ms)	573 (518, 644)	565 (510, 636)	581 (526, 655)
LVEDVi (mL/m^2^)	78.8 (±13.9)	83.8 (±14.7)	74.1 (±11.1)
LVESVi (mL/m^2^)	31.1 (26.3, 36.7)	34.5 (29.5, 40.3)	28.3 (24.5, 32.7)
LVEF (%)	59.5 (±6.1)	57.8 (±6.2)	61.0 (±5.6)
LVSVi (mL/m^2^)	46.6 (±8.3)	48.2 (±9.0)	45.1 (±7.4)
LVMi (g/m^2^)	45.7 (±8.7)	51.1 (±7.9)	40.6 (±5.9)
RVEDVi (mL/m^2^)	83.2 (±15.2)	90.0 (±15.3)	76.9 (±12.1)
RVESVi (mL/m^2^)	35.9 (±9.4)	40.5 (±9.3)	31.5 (±7.1)
RVEF (%)	57.2 (±6.1)	55.1 (±5.9)	59.1 (±5.6)
RVSVi (mL/m^2^)	47.4 (±8.7)	49.5 (±9.3)	45.4 (±7.7)
PDA AoD (10^−3^/mmHg)	2.3 (1.6, 3.1)	2.3 (1.7, 3.1)	2.2 (1.5, 3.0)

Mean (standard deviation) for continuous data, number (percentage) for categorical data. Median (interquartile range) where absolute skew is ≥0.9.

IPAQ, International Physical Activity Questionnaire; i, indexation to body surface area; LVEDVi, left ventricular end-diastolic volume; LVEF, left ventricular ejection fraction; LVM, left ventricular mass; LVESVi, left ventricular end-systolic volume; LVSVi, left ventricular stroke volume; MET, metabolic equivalents; MI, myocardial infarction; PDA AoD, aortic distensibility at the proximal descending aorta; RVEDVi, right ventricular end-diastolic volume; RVEF, right ventricular ejection fraction; RVESVi, right ventricular end-systolic volume; RVSVi, right ventricular stroke volume.

### Association of CMR indices with FI

**Figure 2:**
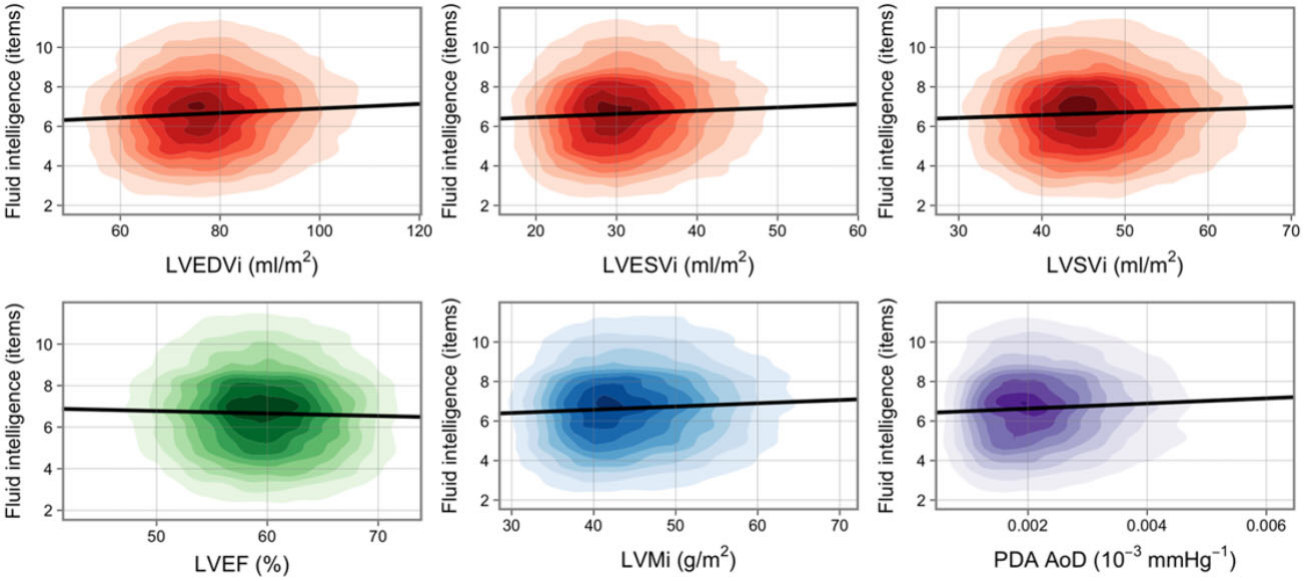
Univariate linear regression models of the association between fluid intelligence and CMR measures. Each graph displays a kernel density plot of one CMR variable against one cognition variable. The nine coloured rings each represent a decile of the data, while the remaining 10% lies in the uncoloured area. Univariate linear regression is shown by black line. All plot areas are trimmed at the 1st and 99th percentile in both *x* and *y* directions. Fluid intelligence has had uniform random jitter/noise (−0.5, 0.5) added for visual smoothing. CMR, cardiovascular magnetic resonance; i, indexation to body surface area; LVEDVi, left ventricular end-diastolic volume; LVEF, left ventricular ejection fraction; LVESVi, left ventricular end-systolic volume; LVSVi, left ventricular stroke volume; PDA AoD, Aortic distensibility at the proximal descending aorta.

In fully adjusted models, higher FI (better cognition) was associated with larger LV volumes in end-diastole and end-systole, higher LV stroke volume, and greater LV mass (*Table 2* and *Figure 2*). The association with LV ejection fraction was not statistically significant. Higher FI was associated with greater aortic distensibility (*Table 2* and *Figure 2*). Higher FI was also associated with larger RV volumes in end-diastole and end-systole, and with larger RV stroke volumes (Supplementary data online, *Table S2*). All associations were consistent for both men and women (*Table 2* and Supplementary data online, *Table S2*). There was no evidence of interaction effect with sex or age in relationships with the LV or RV measures (Supplementary data online, *Table S3*). There was a significant interaction effect with age for the association between FI and aortic distensibility (Supplementary data online, *Table S3*), with participants with higher aortic distensibility showing less rapid age-related decline in FI (Supplementary data online, *Figure S1*).

**Table 2:** Multivariable linear regression models representing standard deviation change in fluid intelligence and reaction time per one standard deviation increase in CMR measures

		Whole cohort	Men	Women
LVEDVi (mL/m^2^)	FI	0.043^a^ (0.031, 0.056)	0.046^a^ (0.030, 0.062)	0.040^a^ (0.020, 0.060)
		1.45 × 10^−11^	3.06 × 10^−8^	9.31 × 10^−5^
	RT	−0.028^a^ (−0.040, −0.015)	−0.031^a^ (−0.047, −0.015)	−0.024^a^ (−0.044, −0.004)
		1.24 × 10^−5^	1.64 × 10^−4^	0.018
LVESVi (mL/m^2^)	FI	0.040^a^ (0.028, 0.053)	0.044^a^ (0.028, 0.059)	0.035^a^ (0.014, 0.055)
		2.76 × 10^−10^	6.28 × 10^−8^	0.001
	RT	−0.019^a^ (−0.031, −0.006)	−0.020^a^ (−0.036, −0.005)	−0.017 (−0.038, 0.004)
		0.003	0.011	0.104
LVEF (%)	FI	−0.018^a^ (−0.030, −0.006)	−0.026^a^ (−0.043, −0.010)	−0.009 (−0.026, 0.008)
		0.003	0.002	0.303
	RT	0.002 (−0.010, 0.014)	0.002 (−0.014, 0.018)	0.002 (−0.015, 0.019)
		0.725	0.831	0.792
LVSVi (mL/m^2^)	FI	0.026^a^ (0.015, 0.038)	0.027^a^ (0.011, 0.043)	0.026^a^ (0.008, 0.044)
		1.17 × 10^−5^	7.70 × 10^−4^	0.004
	RT	−0.024^a^ (−0.035, −0.012)	−0.028^a^ (−0.043, −0.012)	−0.019 (−0.037, −0.001)
		7.81 × 10^−5^	5.03 × 10^−4^	0.039
LVMi (g/m^2^)	FI	0.048^a^ (0.034, 0.063)	0.042^a^ (0.023, 0.060)	0.058^a^ (0.035, 0.081)
		3.50 × 10^−11^	1.09 × 10^−5^	6.87 × 10^−7^
	RT	−0.039^a^ (−0.053, −0.025)	−0.045^a^ (−0.063, −0.027)	−0.032^a^ (−0.055, −0.010)
		8.25 × 10^−8^	1.26 × 10^−6^	0.005
PDA AoD (×10^−3^/mmHg)	FI	0.030^a^ (0.014, 0.045)	0.033^a^ (0.010, 0.057)	0.032^a^ (0.010, 0.053)
		2.02 × 10^−4^	0.006	0.003
	RT	−0.017 (−0.032, −0.001)	−0.016 (−0.039, 0.006)	−0.015 (−0.036, 0.006)
		0.036	0.159	0.171

Results are standardized beta coefficients followed by 95% confidence interval in brackets and corresponding *P*-value displayed below. Each cell represents results from an individual linear regression model. Models are adjusted for: age, sex (whole cohort only), education, deprivation, diabetes, hypertension, high cholesterol, prior myocardial infarction, smoking, alcohol, and exercise. PDA AoD has been scaled to remove skew.

CMR, cardiovascular magnetic resonance; FI, fluid intelligence; i, indexation to body surface area; LVEDVi, left ventricular end-diastolic volume; LVEF, left ventricular ejection fraction; LVESVi, left ventricular end-systolic volume; LVSVi, left ventricular stroke volume; PDA AoD, aortic distensibility at the proximal descending aorta. RT, reaction time.

a *P*-value is significant using a false discovery rate of 5%.

### Association of CMR indices with RT

**Figure 3:**
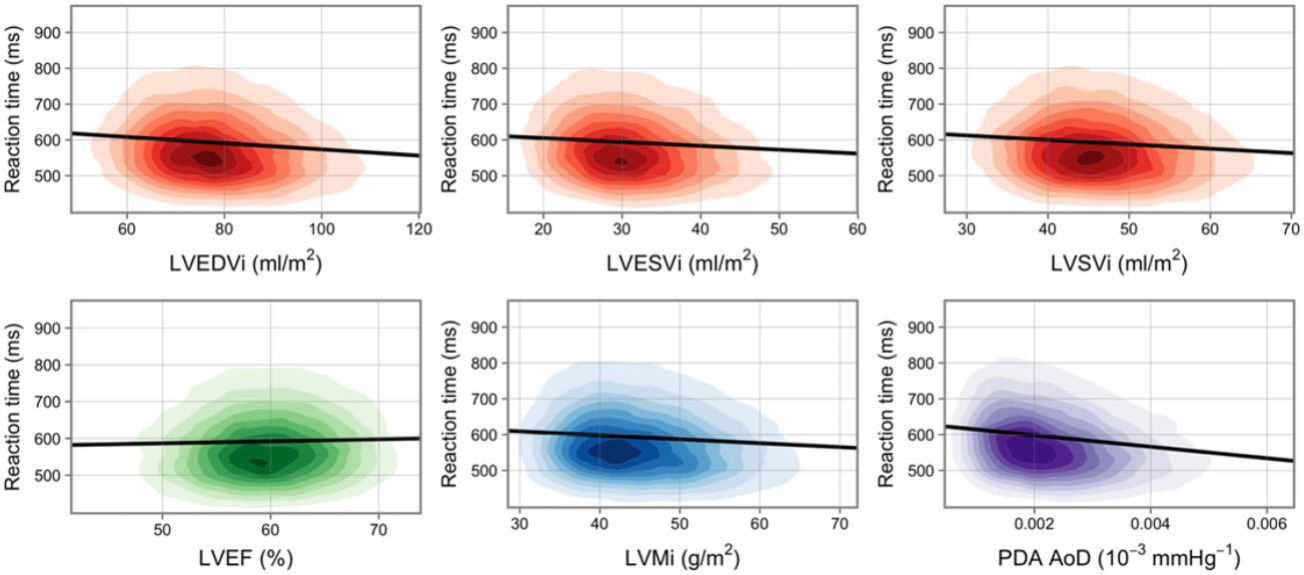
Univariate linear regression models of the association between reaction time and CMR measures. Each graph displays a kernel density plot of one CMR variable against one cognition variable. The nine coloured rings each represent a decile of the data, while the remaining 10% lies in the uncoloured area. Univariate linear regression is shown by black line. All plot areas are trimmed at the 1st and 99th percentile in both *x* and *y* directions. CMR, cardiovascular magnetic resonance; i, indexation to body surface area; LVEDVi, left ventricular end-diastolic volume; LVEF, left ventricular ejection fraction; LVESVi, left ventricular end-systolic volume; LVSVi, left ventricular stroke volume; PDA AoD, Aortic distensibility at the proximal descending aorta.

In fully adjusted models, lower RT (better cognition) was associated with larger LV volumes in end-diastole, higher LV stroke volume, and greater LV mass (*Table 2* and *Figure 3*). Lower RT was also associated with greater aortic distensibility, but this relationship was not statistically significant. Overall, associations were consistent for both men and women (*Table 2*). There was no evidence of interaction effect with sex or age in relationships with the LV or RV measures (Supplementary data online, *Table S3*).

### Non-linearity of relationships

All models were screened for non-linearity with cubic and squared polynomials. For both FI and RT, in fully adjusted models, there was a trend towards attenuation of associations at the high extremes of the distribution for LV volumes and mass (very high volumes and mass). This appeared most convincing for the relationship between FI and LV end-systolic volume, where there was suggestion of attenuation and possible reversal of the direction of association at the very high extremes of the distribution (Supplementary data online, *Figure S2*). However, nested model testing indicated that none of the non-linear models showed a statistically significant improvement over linear model fits (Supplementary data online, *Table S4*).

## Discussion

### Summary of findings

In this predominantly healthy cohort of 15 384 women and 14 379 men from the UK Biobank, we demonstrated association of better cognitive performance with CMR measures likely representing a healthier cardiovascular phenotype, independent of a range of lifestyle, demographic, and vascular risk factors. Specifically, better cognitive performance (higher FI and lower RT) was associated with larger LV and RV volumes, greater LV and RV stroke volumes, higher LV mass, and greater aortic distensibility. There was some evidence of non-linearity for the relationship between FI and LV end-systolic volume, with a trend towards reversal of the direction of association at the high extremes of the distribution (very high volumes). Associations appeared consistent for men and women and with age. For the relationship with FI, there was significant interaction between aortic distensibility and age, with participants with higher aortic distensibility showing less rapid age-related decline in FI.

### Interpretation of cardiovascular phenotypes

Although there was no prerequisite for healthy status for recruitment into UK Biobank, there is a significant healthy participant effect, as such, our results reflect trends within a spectrum of normality. This means that in this analysis, for the most part, we do not report transitions from health to ‘disease’, but rather trends within a predominantly healthy sample. It is also essential that interpretation of the nature of cardiac phenotypes considers the overall pattern of associations as interpretation of single CMR metrics in isolation, outside the context of the other metrics, may be misleading.

Our findings demonstrate association of better cognitive performance with larger ventricular cavity volumes, larger LV and RV stroke volumes, and higher LV mass. This pattern of associations is indicative of better right and left ventricular contractile function (higher stroke volumes) and a pattern of ventricular remodelling, interpreted within the spectrum of normality, akin to decelerated heart ageing (reverse of alterations seen in healthy ageing). There was some evidence of reversal of the direction of associations between FI and LV end-systolic volume at the high extremes of the distribution (very high volumes), suggesting that LV volumes larger than the normal range are linked with poorer cognition. However, within our analysis sample, the non-linear models did not show a statistically significant improvement over linear model fits. This may be because there were few participants with extreme values in our sample. Better cognitive performance was also linked to greater aortic distensibility (statistically significant for FI). Aortic distensibility is a measure of local aortic compliance and a maker of aortic bioelastic function, with higher distensibility values indicating better vascular health. Conversely, poorer cognitive function was associated with smaller ventricular volumes and lower LV mass, together with smaller LV and RV stroke volumes, and lower aortic compliance. Overall, this presents a picture of a cardiac phenotype with poorer myocardial function, small, perhaps stiff, ventricles, and higher aortic stiffness. This suggests that poorer cognition is associated with adverse cardiovascular phenotypes, perhaps resembling a heart failure preserved ejection fraction (HFpEF) phenotype.

### Comparison with existing literature

Existing evidence is limited to small cohorts of select populations with highly variable study designs. Several studies report poorer cognitive function indices in small heart failure cohorts. Zuccalà *et al.*^28^ report an independent association between poorer LV function on echocardiography and worse performance in a number of cognitive tests (mini mental state examination, Raven score) in 57 patients with heart failure. In a study of structural brain abnormalities in heart failure patients, Vogels *et al.*^29^ report greater periventricular and white matter hyperintensities, lacunar and cortical infarcts, and global and medial temporal lobe atrophy in 58 patients with heart failure compared with controls.^30^ Similarly, studies in dementia cohorts demonstrate links with adverse cardiovascular phenotypes. Oh *et al.*^31^ describe a correlation between greater left atrial enlargement on echocardiography (an early indicator of raised filling pressures and diastolic dysfunction) and adverse white matter changes on brain magnetic resonance imaging in 93 patients with dementia. Two other cohort studies demonstrate greater prevalence of diastolic dysfunction (assessed by echocardiography) in individuals with Alzheimer’s disease compared with controls.^32^^,^^33^ Limited studies have examined associations with other cardiovascular phenotypes. In a cohort of 303 participants, Manolio *et al.*^34^ report association of greater cerebral atrophy on brain MRI with greater internal carotid artery thickness on ultrasound (a marker of atherosclerosis risk).

Whilst direct comparisons with our study are not possible, in general, existing work supports associations between adverse cardiovascular phenotypes and poorer cognitive function metrics. In particular, there is evidence to support association of poorer cognitive function indices with heart failure, which is perhaps more pronounced in those with diastolic heart failure.^35^ This is consistent with our findings demonstrating association of poorer cognitive function with smaller LV/RV cavities and lower LV/RV stroke volumes. Overall, this pattern of associations is suggestive of an adverse remodelling phenotype most in keeping with an HFpEF pattern of dysfunction, in which diastolic impairment is a prominent feature.

### Potential underlying mechanisms

**Figure 4:**
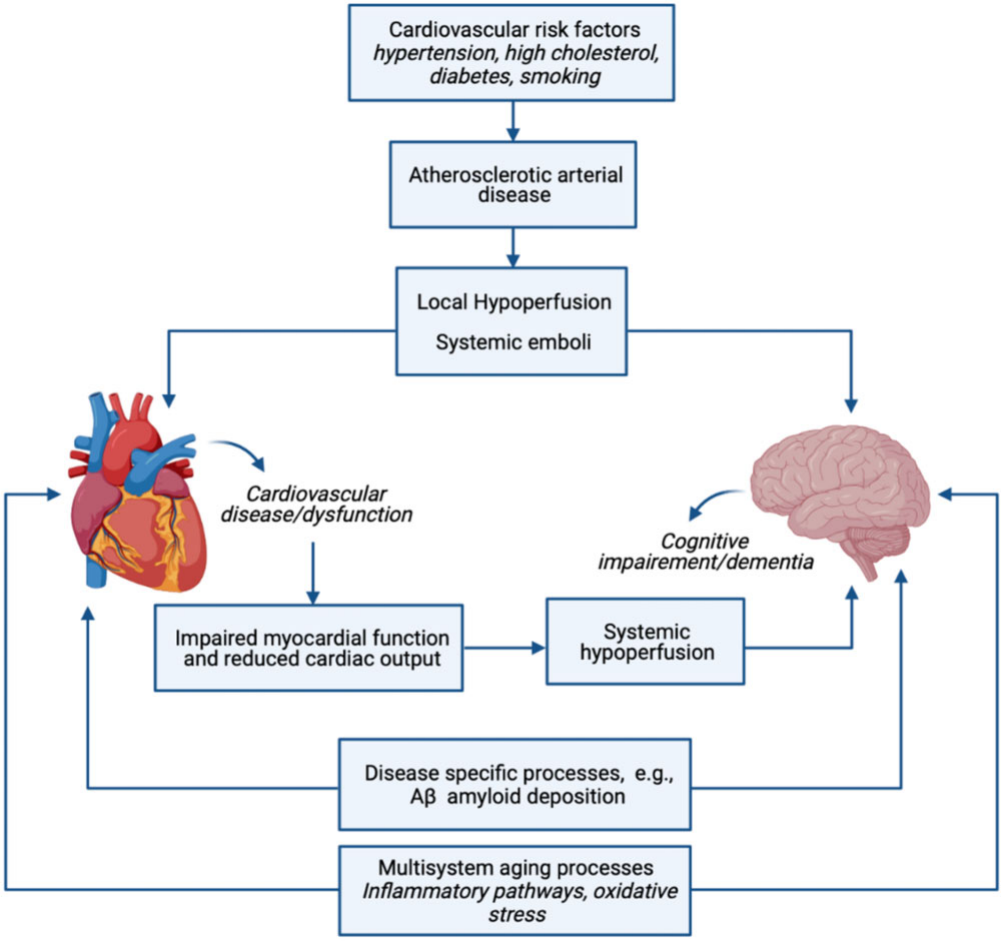
Potential underlying mechanism of heart–brain associations. Figure created with BioRender.com.

Numerous studies highlight links between individual cardiovascular risk factors (diabetes, high cholesterol, smoking, hypertension, and obesity) and worse cognitive performance.^36–40^ Furthermore, association of cardiovascular risk factors and subclinical cardiovascular disease with worse cognition and dementia has been demonstrated in multiple large epidemiological studies.^9^^,^^41^^,^^42^ More specific associations between cardiac risk factors and both vascular and Alzheimer’s disease have also been demonstrated in large cohorts.^7^^,^^43^^,^^44^ The systemic atherosclerotic arterial disease that occurs as a consequence of these vascular risk factors may have direct adverse impact on both cardiovascular and brain health through local hypoperfusion and systemic embolic phenomena (*Figure 4*).

Associations between cognitive function and cardiovascular phenotypes in the present study were not attenuated by adjustment for a wide range of vascular risk factors. This raises the possibility of alternative disease mechanisms contributing to heart–brain associations. For instance, limited studies propose that Aβ deposition, which is hallmark of Alzheimer’s disease, may also be pathologically deposited in the myocardium^33^ producing electrographic and echocardiographic manifestations typical of cardiac amyloid. Cardiac amyloid is characteristically associated with a HFpEF pattern of dysfunction. This is consistent with the cardiac phenotype most consistently linked with cognitive impairment and in keeping also with observations in the present study. However, these phenotypes are not specific to cardiac amyloid and may be seen with a wide range of other exposures. Another possibility is that poorer brain and cardiovascular health may both be a consequence of accelerated multisystem ageing. For instance, persistently elevated inflammatory cytokines, which is a proposed driver of accelerated ageing, has been linked to both cardiovascular disease and Alzheimer’s disease.^45^^,^^46^ Regardless of the underlying cause, it seems likely that these pathways initiate a positive feedback cycle of adverse heart–brain interactions with cardiac dysfunction resulting in chronic systemic hypoperfusion, disruptions to cerebral perfusion, and further exacerbation of brain injuries (*Figure 4*).

Whatever the underlying mechanisms, our findings suggest links between cardiovascular and cognitive health which might, with further investigation and validation, underpin novel clinical approaches to risk assessment for associated outcomes such as myocardial infarction and dementia.

### Strengths and limitations

In this study, we made use of the large and standardized UK Biobank dataset to describe novel associations between cognitive function and CMR phenotypes. The extensive algorithm-coded morbidity, demographic, and lifestyle data available permitted adjustment for a wide range of covariates. However, inherent to the observational cross-sectional study design, the possibility of residual confounding cannot be excluded, and it is not possible to establish a strict causal relationship from the results. Further, the large sample size in this study may reveal statistically but not clinically relevant associations. With this in mind, we have taken a strict hypothesis based approach to the analysis, applied conservative correction of *P*-value thresholds, and consider biological (rather than clinical) interpretation of the findings. Common to all research in the field of cognitive performance and dementia, the questionnaires and scoring systems used to quantify cognitive performance may not accurately reflect global cognitive ability and may be subject to bias depending on underlying educational status and other factors. In addition, there is, as is expected with such cohorts, evidence of healthy selection in UK Biobank,^47^ thus the associations observed in this study describe, predominantly, relationships within the limits of healthy populations. Therefore, the pattern of associations observed may not be directly applicable to disease cohorts. Another limitation of our work is that despite considering the potential confounding effect of an extensive range of exposure variables, we do not identify the underlying mechanism for the observed associations. In a separate analysis, addition of body mass index as covariate to fully adjusted models also did not alter observed associations. Future work dedicated to exploring underlying mechanisms is needed to better understand the links between brain and heart health.

## Conclusions

In this cohort of 29 763 UK Biobank participants, better cognitive performance was associated with CMR and aortic distensibility measures likely representing a healthier cardiovascular phenotype. The associations were in general consistent between men and women and remained robust after adjustment for a range of lifestyle, demographic, and vascular risk factors, implying potential importance of alternative underlying mechanism. These findings thus support links between cardiovascular and cognitive health, inform understanding of associated mechanisms, and suggest a rationale for a cross-system approach to risk assessment for associated disease outcomes.

## Supplementary data

Supplementary data are available at *European Heart Journal - Cardiovascular Imaging* online.

## Funding

This study was conducted using the UK Biobank resource under access application 2964. Z.R.E was supported by a British Heart Foundation Clinical Research Training Fellowship (FS/17/81/33318). S.E.P. acknowledges support from the ‘SmartHeart’ EPSRC programme grant (www.nihr.ac.uk; EP/P001009/1) and also from the CAP-AI programme, London’s first AI enabling programme focused on stimulating growth in the capital’s AI Sector. CAP-AI is led by Capital Enterprise in partnership with Barts Health NHS Trust and Digital Catapult and is funded by the European Regional Development Fund and Barts Charity. N.C.H acknowledges support from the UK Medical Research Council (MRC #405050259 and #U105960371), NIHR Southampton Biomedical Research Centre, University of Southampton, and University Hospital Southampton. S.E.P. and S.N. acknowledge the British Heart Foundation for funding the manual analysis to create a cardiovascular magnetic resonance imaging reference standard for the UK Biobank imaging resource in 5000 CMR scans (www.bhf.org.uk; PG/14/89/31194). S.N., C.M., and A.M. were supported by the Oxford NIHR Biomedical Research Centre and S.N. by the Oxford British Heart Foundation Centre of Research Excellence. E.M.C and N.A. recognize the National Institute for Health Research (NIHR) Integrated Academic Training programme which supports their Academic Clinical Lectureship posts. Ce.M. and S.S. were supported by the NIHR Oxford Health Biomedical Research Centre and the Wellcome Centre for Integrative Neuroimaging. S.S. was funded by a UK Alzheimer’s Society Research Fellowship (Grant 441). L.B. acknowledges support from the British Heart Foundation (PG/15/74/31747).

**Conflict of interest:** none declared.

## Data availability statement

This research was conducted using the UKB resource under access application 2964. UK Biobank will make the data available to all bona fide researchers for all types of health-related research that is in the public interest, without preferential or exclusive access for any persons. All researchers will be subject to the same application process and approval criteria as specified by UK Biobank. For more details on the access procedure, see the UK Biobank website: http://www.ukbiobank.ac.uk/register-apply/.

## Supplementary Material

jeab075_supplementary_data

## References

[1] World Health Organization (WHO). *Ageing and Health*. https://www.who.int/news-room/fact-sheets/detail/ageing-and-health (13 October 2020, date last accessed).

[2] He W , Goodkind D, Kowal P. *International Federation on Aging, An Aging World: 2015*. 2016. https://ifa.ngo/publication/demographics/aging-world-2015/ (28 January 2020, date last accessed).

[3] Qiu C , Fratiglioni L. A major role for cardiovascular burden in age-related cognitive decline. Nat Rev Cardiol 2015;12:267–77. 25583619 10.1038/nrcardio.2014.223

[4] Knopman D , Boland LL, Mosley T, Howard G, Liao D, Szklo M et al Cardiovascular risk factors and cognitive decline in middle-aged adults. Neurology 2001;56:42–8. 11148234 10.1212/wnl.56.1.42

[5] Mielke MM , Rosenberg PB, Tschanz J, Cook L, Corcoran C, Hayden KM et al Vascular factors predict rate of progression in Alzheimer disease. Neurology 2007;69:1850–8. 17984453 10.1212/01.wnl.0000279520.59792.fe

[6] Deschaintre Y , Richard F, Leys D, Pasquier F. Treatment of vascular risk factors is associated with slower decline in Alzheimer disease. Neurology 2009;73:674–80. 19720973 10.1212/WNL.0b013e3181b59bf3

[7] Rusanen M , Kivipelto M, Levälahti E, Laatikainen T, Tuomilehto J, Soininen H et al Heart diseases and long-term risk of dementia and Alzheimer’s disease: a population-based CAIDE study. J Alzheimers Dis 2014;42:183–91. 24825565 10.3233/JAD-132363

[8] Lyall DM , Celis-Morales CA, AJ, Gill JMR, Mackay DF, McIntosh AM et al Associations between single and multiple cardiometabolic diseases and cognitive abilities in 474 129 UK Biobank participants. Eur Heart J 2017;38:584–5. 28363219 10.1093/eurheartj/ehw528 PMC5381595

[9] Cox SR , Lyall DM, Ritchie SJ, Bastin ME, Harris MA, Buchanan CR et al Associations between vascular risk factors and brain MRI indices in UK Biobank. Eur Heart J 2019;40:2290–300. 30854560 10.1093/eurheartj/ehz100 PMC6642726

[10] Raisi-Estabragh Z , Petersen SE. Cardiovascular research highlights from the UK Biobank: opportunities and challenges. Cardiovasc Res 2020;116:e12–15. 31778147 10.1093/cvr/cvz294

[11] *UK Biobank: Protocol for a Large-Scale Prospective Epidemiological Resource*, 2007. https://www.ukbiobank.ac.uk/wp-content/uploads/2011/11/UK-Biobank-Protocol.pdf (13 December 2020, date last accessed).

[12] UK Biobank Imaging Study. https://imaging.ukbiobank.ac.uk/ (11 December 2020, date last accessed).

[13] Raisi-Estabragh Z , Harvey NC, Neubauer S, Petersen SE. Cardiovascular magnetic resonance imaging in the UK Biobank: a major international health research resource. Eur Hear J Cardiovasc Imaging 2021;22:251–8. 10.1093/ehjci/jeaa297 PMC7899275 33164079

[14] Hagenaars SP , Harris SE, Davies G, Hill WD, Liewald DCM, Ritchie SJ et al; CHARGE Consortium Pulmonary Group, CHARGE Consortium Aging and Longevity Group. Shared genetic aetiology between cognitive functions and physical and mental health in UK Biobank (N=112 151) and 24 GWAS consortia. Mol Psychiatry 2016;21:1624–32. 26809841 10.1038/mp.2015.225 PMC5078856

[15] Fawns-Ritchie C , Deary IJ. Reliability and validity of the UK Biobank cognitive tests. PLoS One 2020;15:e0231627. 32310977 10.1371/journal.pone.0231627 PMC7170235

[16] UK Biobank Touch-Screen Fluid Intelligence Test, 2012. https://biobank.ndph.ox.ac.uk/showcase/showcase/docs/Fluidintelligence.pdf (20 February 2021, date last accessed).

[17] Gaddis ML , Gaddis GM. Introduction to biostatistics: part 1, basic concepts. Ann Emerg Med 1990;19:86–9. 2297161 10.1016/s0196-0644(05)82149-3

[18] Biobank Touch-Screen Test of Reaction Time (Snap), 2015. https://biobank.ndph.ox.ac.uk/showcase/showcase/docs/Snap.pdf (20 February 2021, date last accessed).

[19] Petersen SE , Matthews PM, Francis JM, Robson MD, Zemrak F, Boubertakh R et al UK Biobank’s cardiovascular magnetic resonance protocol. J Cardiovasc Magn Reson 2016;18:8. 26830817 10.1186/s12968-016-0227-4 PMC4736703

[20] Attar R , Pereañez M, Gooya A, Albà X, Zhang L, de Vila MH et al Quantitative CMR population imaging on 20,000 subjects of the UK Biobank imaging study: LV/RV quantification pipeline and its evaluation. Med Image Anal 2019;56:26–42. 31154149 10.1016/j.media.2019.05.006

[21] Biasiolli L , Hann E, Lukaschuk E, Carapella V, Paiva JM, Aung N et al Automated localization and quality control of the aorta in cine CMR can significantly accelerate processing of the UK Biobank population data. PLoS One 2019;14:e0212272. 30763349 10.1371/journal.pone.0212272 PMC6375606

[22] R Core Team. R: A Language and Environment for Statistical Computing. Vienna, Austria: R Foundation for Statistical Computing; 2019.

[23] RStudio*: Integrated Development for R*. Boston, MA: RStudio, Inc. https://rstudio.com/ (18 October 2020, date last accessed).

[24] Benjamini Y , Hochberg Y. Controlling the false discovery rate: a practical and powerful approach to multiple testing. Source J R Stat Soc Ser B 1995;57:289–300.

[25] Townsend P , Phillimore P, Beattie A. Health and deprivation: inequality and the North. Nurs Stand 1988;2:34. 10.7748/ns.2.17.34.s66 27415096

[26] Craig CL , Marshall AL, Sjöström M, Bauman AE, Booth ML, Ainsworth BE et al International physical activity questionnaire: 12-country reliability and validity. Med Sci Sports Exerc 2003;35:1381–1395. 12900694 10.1249/01.MSS.0000078924.61453.FB

[27] Schnier C , Sudlow CU. *Biobank Algorithmically-Defined Health Outcomes (Chief Scientist), with Input From Members of the UK Biobank Follow-up and Outcomes Adjudication Group*, 2017. https://biobank.ctsu.ox.ac.uk/crystal/crystal/docs/alg_outcome_main.pdf (27 March 2020, date last accessed).

[28] Zuccalà G , Cattel C, Manes-Gravina E, Niro MG, Di Cocchi A, Bernabei R. Left ventricular dysfunction: a clue to cognitive impairment in older patients with heart failure. J Neurol Neurosurg Psychiatry 1997;63:509–512. 9343133 10.1136/jnnp.63.4.509 PMC2169754

[29] Vogels RLC , van der Flier WM, van Harten B, Gouw AA, Scheltens P, Schroeder-Tanka JM et al Brain magnetic resonance imaging abnormalities in patients with heart failure. Eur J Heart Fail 2007;9:1003–1009. 17719270 10.1016/j.ejheart.2007.07.006

[30] Jefferson AL. Cardiac output as a potential risk factor for abnormal brain aging. J Alzheimers Dis 2010;20:813–821. 20413856 10.3233/JAD-2010-100081 PMC3041147

[31] Oh JE , Shin JW, Sohn EH, Jung JO, Jeong SH, Song HJ et al Effect of cardiac function on cognition and brain structural changes in dementia. J Clin Neurol 2012;8:123–129. 22787496 10.3988/jcn.2012.8.2.123 PMC3391617

[32] Sanna GD , Nusdeo G, Piras MR, Forteleoni A, Murru MR, Saba PS et al Cardiac abnormalities in Alzheimer disease: clinical relevance beyond pathophysiological rationale and instrumental findings? JACC Heart Fail 2019;7:121–128. 30704603 10.1016/j.jchf.2018.10.022

[33] Troncone L , Luciani M, Coggins M, Wilker EH, Ho CY, Codispoti KE et al Aβ amyloid pathology affects the hearts of patients with Alzheimer’s disease: mind the heart. J Am Coll Cardiol 2016;68:2395–2407. 27908343 10.1016/j.jacc.2016.08.073 PMC5142757

[34] Manolio TA , Kronmal RA, Burke GL, Poirier V, O'Leary DH, Gardin JM et al Magnetic resonance abnormalities and cardiovascular disease in older adults the cardiovascular health study. Stroke 1994;25:318–327. 8303738 10.1161/01.str.25.2.318

[35] Suwa M , Ito T. Correlation between cognitive impairment and left ventricular diastolic dysfunction in patients with cardiovascular diseases. Int J Cardiol 2009;136:351–354. 18684536 10.1016/j.ijcard.2008.04.099

[36] Lu FP , Lin KP, Kuo HK. Diabetes and the risk of multi-system aging phenotypes: a systematic review and meta-analysis. PLoS One 2009;4:e4144. 19127292 10.1371/journal.pone.0004144 PMC2607544

[37] Peters R , Poulter R, Warner J, Beckett N, Burch L, Bulpitt C. Smoking, dementia and cognitive decline in the elderly, a systematic review. BMC Geriatr 2008;8:36. 19105840 10.1186/1471-2318-8-36 PMC2642819

[38] Anstey KJ , Lipnicki DM, Low L-F. Cholesterol as a risk factor for dementia and cognitive decline: a systematic review of prospective studies with meta-analysis. Am J Geriatr Psychiatry 2008;16:343–354. 18448847 10.1097/JGP.0b013e31816b72d4

[39] Novak V , Hajjar I. The relationship between blood pressure and cognitive function. Nat Rev Cardiol 2010;7:686–698. 20978471 10.1038/nrcardio.2010.161 PMC3328310

[40] Anstey KJ , Cherbuin N, Budge M, Young J. Body mass index in midlife and late-life as a risk factor for dementia: a meta-analysis of prospective studies. Obes Rev 2011;12:e426–e437. 21348917 10.1111/j.1467-789X.2010.00825.x

[41] Wolf PA. Contributions of the framingham heart study to stroke and dementia epidemiologic research at 60 years. Arch Neurol 2012;69:567–571. 22213410 10.1001/archneurol.2011.977 PMC3380159

[42] Chaves PHM , Kuller LH, O'Leary DH, Manolio TA, Newman AB; Cardiovascular Health Study. Subclinical cardiovascular disease in older adults: insights from the Cardiovascular Health Study. Am J Geriatr Cardiol 2004;13:137–149. 15133417 10.1111/j.1076-7460.2004.02120.x

[43] Gelber RP , Launer LJ, White LR. The Honolulu-Asia Aging Study: epidemiologic and neuropathologic research on cognitive impairment. Curr Alzheimer Res 2012;9:664–672. 22471866 10.2174/156720512801322618 PMC4795939

[44] Fratiglioni L , Winblad B, von Strauss E. Prevention of Alzheimer’s disease and dementia. Major findings from the Kungsholmen Project. Physiol Behav 2007;92:98–104. 17588621 10.1016/j.physbeh.2007.05.059

[45] Soysal P , Arik F, Smith L, Jackson SE, Isik AT. Inflammation, frailty and cardiovascular disease. Adv Exp Med Biol 2020;1216:55–64. 31894547 10.1007/978-3-030-33330-0_7

[46] Irwin MR , Vitiello MV. Implications of sleep disturbance and inflammation for Alzheimer’s disease dementia. Lancet Neurol 2019;18:296–306. 30661858 10.1016/S1474-4422(18)30450-2

[47] Batty GD , Gale CR, Kivimäki M, Deary IJ, Bell S. Comparison of risk factor associations in UK Biobank against representative, general population based studies with conventional response rates: prospective cohort study and individual participant meta-analysis. BMJ 2020;368:1–8. 10.1136/bmj.m131 PMC7190071 32051121

