# Supplementary material for: Associations of cognitive performance with cardiovascular magnetic resonance phenotypes in the UK Biobank
Source: Eur Heart J Cardiovasc Imaging. 2021 May 14;23(5):663–72. doi: 10.1093/ehjci/jeab075 (PMC9016359; doi:10.1093/ehjci/jeab075)

**Supplementary Figure 1. Interaction effect between aortic distensibility and age in the relationship with fluid intelligence**


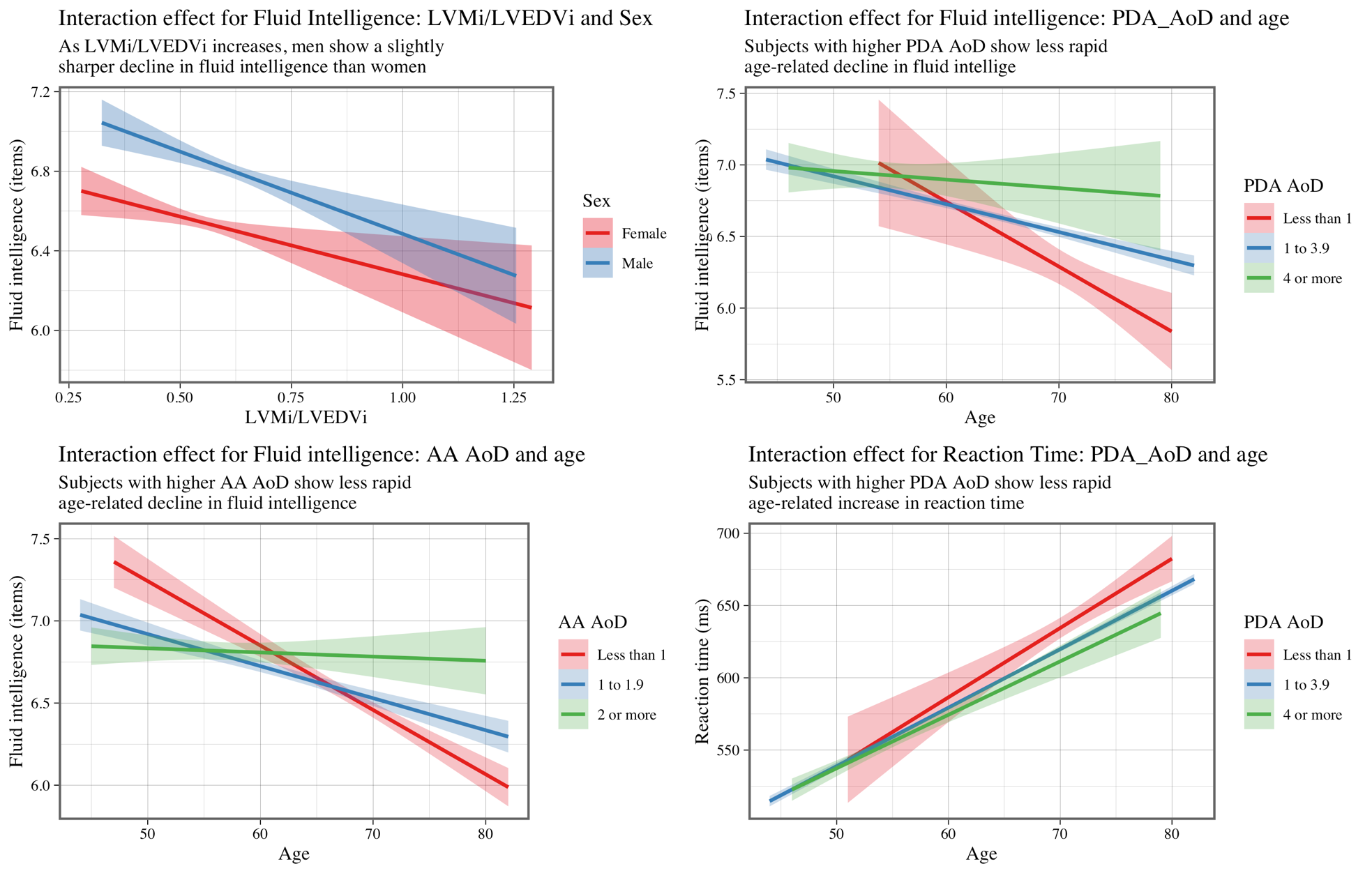


**Supplementary Figure 1 footnote:** participants with higher distensibility show less rapid age-related decline in fluid intelligence. PDA AoD: aortic distensibility at the ascending aorta. PDA AoD units are x10^-3^ mmHg^-1^.

**Supplementary Figure 2. Fully adjusted linear (blue) and polynomial (red) models of associations between fluid intelligence (A) and reaction time (B) with CMR measures**


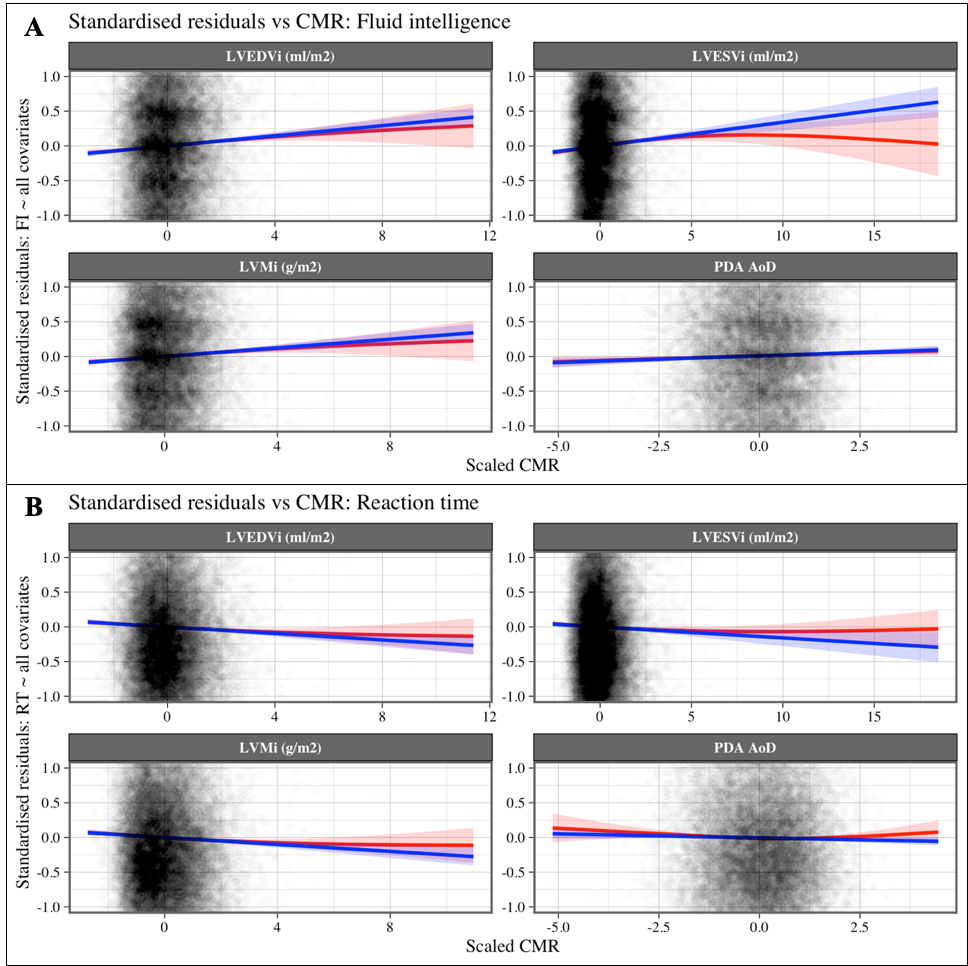

Supplement: jeab075_supplementary_data [file jeab075_supplementary_data.zip › cognition_sFigs_R1.docx]
