# Supplementary material for: Associations of cognitive performance with cardiovascular magnetic resonance phenotypes in the UK Biobank
Source: Eur Heart J Cardiovasc Imaging. 2021 May 14;23(5):663–72. doi: 10.1093/ehjci/jeab075 (PMC9016359; doi:10.1093/ehjci/jeab075)
Supplement: jeab075_supplementary_data [file jeab075_supplementary_data.zip › cognition_sTables_R1.docx]

**Supplementary Table 1. Fluid intelligence assessment questions**

| Question | Multiple choice options |
| --- | --- |
| Add the following numbers together: 1 2 3 4 5, is the answer? | 13, 14, 15, 16, 17, Do not know, Prefer not to answer |
| Which number is the largest? | 642, 308, 987, 714, 253, Do not know, Prefer not to answer |
| Bud is to flower as child is to? | Grow, Develop, Improve, Adult, Old, Do not know, Prefer not to answer |
| 11 12 13 14 15 16 17 18  Divide the sixth number to the right of twelve by three. Is the answer? | 5, 6, 7, 8, Do not know, Prefer not to answer |
| If Truda’s mother’s brother is Tim’s sister’s father, what relation is Truda to Tim? | Aunt, Sister, Niece, Cousin, No relation, Do not know, Prefer not to answer |
| If sixty is more than half of seventy-five, multiply twenty- three by three. If not subtract 15 from eighty-five. Is the answer? | 68, 69, 70, 71, 72, Do not know, Prefer not to answer |
| Stop means the same as? | Pause, Close, Cease, Break, Rest, Do not know, Prefer not to answer |
| If David is twenty-one and Owen is nineteen and Daniel is nine years younger than David, what is half their combined age? | 25, 26, 27, 28, 29, Do not know, Prefer not to answer |
| Age is to years as height is to? | Long, Deep, Top, Metres, Tall, Do not know, Prefer not to answer |
| 150...137...125...114...104... What comes next? | 96, 95, 94, 93, 92, Do not know, Prefer not to answer |
| Relaxed means the opposite of? | Calm, Anxious, Cool, Worried, Tense, Do not know, Prefer not to answer |
| 100...99...95...86...70... What comes next? | 50, 49, 48, 47, 46, 45, Do not know, Prefer not to answer |
| If some flinks are plinks and some plinks are stinks then some flinks are definitely stinks? | False, True, Neither true nor false, Not sure, Do not know, Prefer not to answer |

.

**Supplementary Table 2. Multivariable linear regression models representing standard deviation change in fluid intelligence and reaction time per one standard deviation increase in CMR measures***

|  |  | **Whole cohort** | **Men** | **Women** |
| --- | --- | --- | --- | --- |
| RVEDVi (ml/m2) | FI | 0.072* [0.059, 0.085] | 0.070* [0.052, 0.087] | 0.075* [0.055, 0.096] |
|  |  | 4.24x10^-27^ | 2.98x10^-15^ | 3.31x10^-13^ |
|  | RT | -0.036* [-0.049, -0.023] | -0.038* [-0.055, -0.021] | -0.035* [-0.056, -0.015] |
|  |  | 5.26x10^-8^ | 1.25x10^-5^ | 6.94x10^-4^ |
| RVESVi (ml/m2) | FI | 0.079* [0.066, 0.093] | 0.072* [0.055, 0.089] | 0.091* [0.070, 0.112] |
|  |  | 1.01x10^-31^ | 4.92x10^-16^ | 1.52x10^-17^ |
|  | RT | -0.035* [-0.049, -0.022] | -0.035* [-0.053, -0.018] | -0.038* [-0.058, -0.017] |
|  |  | 1.50x10^-7^ | 5.13x10^-5^ | 4.59x10^-4^ |
| RVEF (%) | FI | -0.039* [-0.050, -0.027] | -0.033* [-0.050, -0.016] | -0.045* [-0.062, -0.028] |
|  |  | 2.76x10^-10^ | 1.60x10^-4^ | 1.76x10^-7^ |
|  | RT | 0.012 [-0.000, 0.024] | 0.011 [-0.006, 0.028] | 0.013 [-0.004, 0.030] |
|  |  | 0.052 | 0.195 | 0.136 |
| RVSVi (ml/m2) | FI | 0.035* [0.024, 0.047] | 0.038* [0.022, 0.054] | 0.031* [0.013, 0.049] |
|  |  | 5.83x10^-9^ | 2.70x10^-6^ | 6.80x10^-4^ |
|  | RT | -0.022* [-0.033, -0.010] | -0.025* [-0.040, -0.009] | -0.019 [-0.037, -0.000] |
|  |  | 3.49x10^-4^ | 0.002 | 0.044 |

**Supplementary Table 2 footnote.** Results are standardised beta coefficients with 95% confidence interval and p-value. An asterisk indicates where the p-value is significant using a false discovery rate of 5%. Each cell represents results from an individual linear regression model. Covariates included: age, sex (whole cohort only), education, deprivation, diabetes, hypertension, hypercholesterolaemia, prior myocardial infarction, smoking, alcohol, exercise. RVEDVi: right ventricular end-diastolic volume; RVEF: right ventricular ejection fraction; RVESVi: right ventricular end-systolic volume; RVSVi: right ventricular stroke volume. i denotes indexation to body surface area.

**Supplementary Table 3. Interaction effects between CMR and age, and between CMR and sex for fluid intelligence in fully adjusted models**

| **Interaction term** | **CMR measure** | **Fluid intelligence (p-value)** | **Reaction time  (p-value)** |
| --- | --- | --- | --- |
| CMR with age | LVEDVi (ml/m^2^) | 0.8721 | 0.6148 |
|  | LVESVi (ml/m^2^) | 0.6195 | 0.7075 |
|  | LVEF (%) | 0.8935 | 0.5582 |
|  | LVSVi (ml/m^2^) | 0.7148 | 0.7223 |
|  | LVMi (g/m^2^) | 0.9119 | 0.0828 |
|  | RVEDVi (ml/m^2^) | 0.7007 | 0.1755 |
|  | RVESVi (ml/m^2^) | 0.6907 | 0.0378 |
|  | RVEF (%) | 0.0410 | 0.1080 |
|  | RVSVi (ml/m^2^) | 0.4968 | 0.891 |
|  | PDA AoD (10^-3^ mmHg^-1^) | 7.09x10^-4^ * | 0.0109 * |
| CMR with sex | LVEDVi (ml/m^2^) | 0.2904 | 0.9246 |
|  | LVESVi (ml/m^2^) | 0.2668 | 0.8309 |
|  | LVEF (%) | 0.1278 | 0.8687 |
|  | LVSVi (ml/m^2^) | 0.5094 | 0.8088 |
|  | LVMi (g/m^2^) | 0.4451 | 0.6434 |
|  | RVEDVi (ml/m^2^) | 0.8876 | 0.7219 |
|  | RVESVi (ml/m^2^) | 0.3882 | 0.5442 |
|  | RVEF (%) | 0.4180 | 0.8233 |
|  | RVSVi (ml/m^2^) | 0.2858 | 0.9470 |
|  | PDA AoD (10^-3^ mmHg^-1^) | 0.0548 | 0.3882 |

**Supplementary Table 3 footnote**: Models adjusted for: age, sex, education, deprivation, diabetes, hypertension, hypercholesterolaemia, prior myocardial infarction, smoking, alcohol, exercise. AoD PDA: aortic distensibility at the proximal descending aorta; LVEDVi: left ventricular end-diastolic volume; LVEF: left ventricular ejection fraction; LVESVi: left ventricular end-systolic volume; RVEDVi: right ventricular end-diastolic volume; RVEF: right ventricular ejection fraction; RVESVi: right ventricular end-systolic volume; LVSVi: left ventricular stroke volume; RVSVi: right ventricular stroke volume. i denotes indexation to body surface area. An asterisk indicates where the p-value is significant using a false discovery rate of 5%. **Supplementary Table 4. Comparison tests between linear and non-linear models**

|  | Fluid intelligence | | Reaction time | |
| --- | --- | --- | --- | --- |
| CMR | Linear vs 2d polynomial | Linear vs 3d polynomial | Linear vs 2d polynomial | Linear vs 3d polynomial |
| LVEDVi (ml/m2) | 0.4956 | 0.2325 | 0.0991 | 0.1555 |
| LVESVi (ml/m2) | 0.0076 | 0.0233 | 0.0495 | 0.1251 |
| LVEF (%) | 0.2230 | 0.4714 | 0.4174 | 0.6345 |
| LVSVi (ml/m2) | 0.5845 | 0.2343 | 0.6092 | 0.4265 |
| LVMi (g/m2) | 0.7945 | 0.8490 | 0.0351 | 0.1069 |
| RVEDVi (ml/m2) | 0.4312 | 0.5715 | 0.7481 | 0.8136 |
| RVESVi (ml/m2) | 0.3604 | 0.4604 | 0.8565 | 0.8858 |
| RVEF (%) | 0.0526 | 0.1496 | 0.5481 | 0.8097 |
| RVSVi (ml/m2) | 0.2767 | 0.2204 | 0.9821 | 0.8759 |
| PDA AoD | 0.8463 | 0.8409 | 0.0078 | 0.0020 |

**Supplementary Table 4 footnote:** Numbers are p-values, (pr(F)) from analysis of variance tests for nested models. None of the p-values above were significant using a false discovery rate of 5%. Significance threshold is p<0.0013. PDA AoD: aortic distensibility at the proximal descending aorta; LVEDVi: left ventricular end-diastolic volume; LVEF: left ventricular ejection fraction; LVESVi: left ventricular end-systolic volume; RVEDVi: right ventricular end-diastolic volume; RVEF: right ventricular ejection fraction; RVESVi: right ventricular end-systolic volume; LVSVi: left ventricular stroke volume; RVSVi: right ventricular stroke volume. i denotes indexation to body surface area.

**Supplementary Table 5. Fully adjusted multivariable models with non-standardised coefficients, Change in cognition variable associated with a one unit increase in CMR variable.**

| **CMR** | **Fluid intelligence** | **Reaction time** |
| --- | --- | --- |
| LVEDVi (ml/m2) | 0.006* [0.005, 0.008] | -0.218* [-0.315, -0.120] |
|  | 1.45x10^-11^ [0.1056] | 1.24x10^-5^ [0.0986] |
| LVESVi (ml/m2) | 0.010* [0.007, 0.013] | -0.235* [-0.390, -0.079] |
|  | 2.76x10^-10^ [0.1054] | 0.0031 [0.0983] |
| LVEF (%) | -0.006* [-0.010, -0.002] | 0.037 [-0.171, 0.245] |
|  | 0.0026 [0.1043] | 0.7254 [0.0979] |
| LVSVi (ml/m2) | 0.007* [0.004, 0.009] | -0.309* [-0.463, -0.156] |
|  | 1.17x10^-5^ [0.1048] | 7.81x10^-5^ [0.0985] |
| LVMi (g/m2) | 0.011* [0.008, 0.015] | -0.485* [-0.663, -0.308] |
|  | 3.50x10^-11^ [0.1056] | 8.25x10^-8^ [0.0989] |
| PDA AoD x 1000 (log) | 0.133* [0.063, 0.203] | -3.898 [-7.538, -0.257] |
|  | 2.02x10^-4^ [0.1047] | 0.0359 [0.0987] |
| RVEDVi (ml/m2) | 0.010* [0.008, 0.012] | -0.259* [-0.352, -0.166] |
|  | 4.24x10^-27^ [0.1079] | 5.26x10^-8^ [0.0991] |
| RVESVi (ml/m2) | 0.018* [0.015, 0.020] | -0.412* [-0.565, -0.258] |
|  | 1.01x10^-31^ [0.1086] | 1.50x10^-7^ [0.0991] |
| RVEF (%) | -0.013* [-0.017, -0.009] | 0.209 [-0.002, 0.420] |
|  | 2.76x10^-10^ [0.1053] | 0.0522 [0.0982] |
| RVSVi (ml/m2) | 0.008* [0.006, 0.011] | -0.270* [-0.418, -0.122] |
|  | 5.83x10^-9^ [0.1052] | 3.49x10^-4^ [0.0986] |

**Supplementary Table 5 footnote:** Result format – point estimate, [95% CI], p-value, [R^2^]. AoD PDA: aortic distensibility at the proximal descending aorta; LVEDVi: left ventricular end-diastolic volume; LVEF: left ventricular ejection fraction; LVESVi: left ventricular end-systolic volume; RVEDVi: right ventricular end-diastolic volume; RVEF: right ventricular ejection fraction; RVESVi: right ventricular end-systolic volume; LVSVi: left ventricular stroke volume; RVSVi: right ventricular stroke volume. i denotes indexation to body surface area.
